# Diffusion Tensor Imaging (DTI) Correlates of Self-Reported Sleep Quality and Depression Following Mild Traumatic Brain Injury

**DOI:** 10.3389/fneur.2018.00468

**Published:** 2018-06-20

**Authors:** Adam C. Raikes, Sahil Bajaj, Natalie S. Dailey, Ryan S. Smith, Anna Alkozei, Brieann C. Satterfield, William D. S. Killgore

**Affiliations:** Social, Cognitive, and Affective Neuroscience Laboratory, Department of Psychiatry, College of Medicine, University of Arizona, Tucson, AZ, United States

**Keywords:** white matter integrity, Pittsburgh sleep quality index, beck depression inventory, fractional anisotropy, radial diffusivity, internal capsule, superior fronto-occipital fasciculus, corona radiata

## Abstract

**Background:** Mild traumatic brain injuries (mTBIs) are a significant social, sport, and military health issue. In spite of advances in the clinical management of these injuries, the underlying pathophysiology is not well-understood. There is a critical need to advance objective biomarkers, allowing the identification and tracking of the long-term evolution of changes resulting from mTBI. Diffusion-weighted imaging (DWI) allows for the assessment of white-matter properties in the brain and shows promise as a suitable biomarker of mTBI pathophysiology.

**Methods:** 34 individuals within a year of an mTBI (age: 24.4 ± 7.4) and 18 individuals with no history of mTBI (age: 23.2 ± 3.4) participated in this study. Participants completed self-report measures related to functional outcomes, psychological health, post-injury symptoms, and sleep, and underwent a neuroimaging session that included DWI. Whole-brain white matter was skeletonized using tract-based spatial statistics (TBSS) and compared between groups as well as correlated within-group with the self-report measures.

**Results:** There were no statistically significant anatomical differences between the two groups. After controlling for time since injury, fractional anisotropy (FA) demonstrated a negative correlation with sleep quality scores (higher FA was associated with better sleep quality) and increasing depressive symptoms in the mTBI participants. Conversely, mean (MD) and radial diffusivity (RD) demonstrated positive correlations with sleep quality scores (higher RD was associated with worse sleep quality) and increasing depressive symptoms. These correlations were observed bilaterally in the internal capsule (anterior and posterior limbs), corona radiata (anterior and superior), fornix, and superior fronto-occipital fasciculi.

**Conclusion:** The results of this study indicate that the clinical presentation of mTBI, particularly with respect to depression and sleep, is associated with reduced white-matter integrity in multiple areas of the brain, even after controlling for time since injury. These areas are generally associated not only with sleep and emotion regulation but also cognition. Consequently, the onset of depression and sleep dysfunction as well as cognitive impairments following mTBI may be closely related to each other and to white-matter integrity throughout the brain.

## Introduction

Concussions and mild traumatic brain injuries (mTBI) represent a significant public and military health crisis. 1.6 to 3.8 million sport-related concussions (SRCs) are reported annually (1, 2), and more than 300,000 mTBIs (hereafter referring to both mTBIs and SRCs) have been documented in military personnel since the year 2000 (3). The actual incidence of these injuries is likely much higher, as estimates reflect only those for which treatment is sought (4). Treatment costs for mTBIs top $22 billion annually in the United States (5). Individuals sustaining an mTBI may exhibit any number of clinical features, including changes in cognitive and motor function as well as post-injury depression, somatic symptoms, and sleep-wake cycle disruption (6). However, these clinical signs and symptoms are not generally associated with visible structural abnormalities when using traditional diagnostic/clinical neuroimaging techniques (e.g., structural magnetic resonance imaging (MRI) or computed tomography (CT) in the emergency department). Furthermore, although many of the clinical signs and symptoms resolve within the first month post-injury (6), many individuals continue to experience symptoms well beyond this clinical timeframe.

Among those persistent symptoms, sleep disruption and depression are among the most common. Estimates of the prevalence of sleep disruption following mTBI ranges from 30 to 80% (7–9), with complaints of insomnia, hypersomnia, and pleiosomnia all reported (8, 10–12). Individuals with prior mTBI also often report and exhibit depressive symptoms, with an estimated 6% per year being clinically diagnosed with depression (13) and many more exhibiting depressive symptoms (14). Notably, depression may additionally cause altered sleep patterns (15). Collectively, both sleep disruption and depression can impair cognitive and physical function (16–20) and may therefore exacerbate the symptoms of and delay the recovery from an mTBI. However, to date, there are have a limited number of studies that have identified the neural correlates of both sleep disruption (21) or depression (22, 23) following mTBI. Consequently, it is needful to identify objective biomarkers of both the pathophysiology and post-injury recovery that underpin the evolution of post-mTBI sleep disruption and depression.

One imaging methodology that is particularly sensitive to altered brain structure is diffusion tensor imaging (DTI). In DTI, water molecule diffusion properties in the brain are quantified, principally by fractional anisotropy (FA) and mean diffusivity (MD). FA quantifies molecular diffusion along three dimensions (FA = 0: diffusion is equally likely in any direction; FA = 1: diffusion occurs along one direction), while MD quantifies the average three-dimensional diffusion rate. Additionally, radial diffusivity (RD) and axial diffusivity (AD) reflect the rates of diffusion perpendicular and parallel to the underlying tissue, respectively. These diffusion metrics, and most prevalently FA, are thought to provide an index of white matter integrity. Mouse models of neural trauma have demonstrated decreased AD concomitant with axonal damage (24, 25) and negative correlations between RD and myelination [e.g., higher RD is associated with reduced myelination; (25, 26)]. MD, the average of AD and RD, is non-specific with respect to the direction of diffusion. Increases in MD and coincident decreases in FA are often associated with neural trauma and neurodegeneration (27, 28), including mTBI (29–31).

Mild traumatic brain injuries may reflect a model of diffuse axonal injury (DAI), characterized by damage to, and subsequently the loss of, axons, myelin, or both (32–34). Demyelination has been observed in animal models of mTBI (35) and may be secondary to axonal loss or loss of oligodendrocytes supplying undamaged axons (36–39). Regardless of mechanism, white matter integrity may be compromised following mTBI. Therefore, DTI metrics may provide a suitable biomarker of both microstructural changes following mTBI as well as clinical presentation.

With respect to mTBI, numerous publications have featured DTI-related findings in civilian, military, and sport populations, spanning timeframes from acute to remote (years) post-injury (40–42). Despite the density of publications, there is little consistency in the findings with respect to directional changes in DTI metrics. While some studies, for instance, report lower FA following mTBI (43–47), this is not always the case (48–51). Such inconsistent patterns are also present with respect to MD, AD, and RD (43, 46, 48, 52). Given the heterogeneous nature of mechanistic/neural changes in mTBI and generally small study sample sizes, such inconsistency is not unexpected and necessitates additional exploration.

Despite the inconsistency in directional findings for DTI diffusion metrics following mTBI, several affected white matter pathways do exhibit some consistency. Changes or differences in FA and MD in the corpus callosum, anterior and posterior corona radiata, anterior and posterior thalamic radiations, superior and inferior longitudinal fasciculi, corticospinal tracts, and internal capsule, are commonly reported (40, 41, 53). Such consistency of reporting suggests that these white matter tracts may be particularly susceptible to the multiple mechanisms (focal injuries, shearing) that may result in an mTBI (40, 41, 53). Importantly, prior work related to major depressive disorder (54) and insomnia (55, 56), as well as sleep quality (57) and variability (58) in generally healthy populations, has consistently demonstrated correlations with FA, such that more negative outcomes (e.g., greater depressive symptoms, lower sleep quality, and increased variability) are associated with lower FA in these same tracts. Given the overlapping tracts identified on DTI following mTBI and those related to sleep quality and depression from other populations, it is likely that these tracts play an important role in post-mTBI symptom presentation. However, to date, there are no DTI-related findings specific to mTBI and sleep quality or depression.

The purpose of this study was to use DTI to correlate diffusion metrics with self-report indices of sleep quality and depression in individuals with a recent mTBI. This study is part of a larger, on-going project aimed at identifying structural and neural correlates of self-report, neurocognitive, and behavioral outcomes following mTBI. Here, we compared DTI metrics between individuals within a year of an mTBI and individuals with no self-reported history of mTBI. Additionally, we computed within-group correlations between diffusion metrics and self-report outcomes in the National Institute of Neurological Disorders and Stroke (NINDS) Common Data Elements (CDEs; http://www.commondataelements.ninds.nih.gov/) psychiatric and psychological status [e.g., depression; (59)] domain and self-reported sleep quality (60). We hypothesized, consistent with the view that mTBI reflects aspects of DAI (32–34), that FA would be lower, and MD, AD, and RD higher in the mTBI group than in the healthy controls. Further we hypothesized that this pattern would extend to symptom presentation, with greater post-mTBI depressive symptoms, and lower sleep quality associated with lower FA and greater MD, AD, and RD.

## Materials and methods

### Participants

A total of 52 individuals (*n* = 34 with a history of a recent (within 12 months) mTBI; *n* = 18 healthy control individuals with no documented history of head trauma) participated in the present study. Participants were recruited from the Tucson metropolitan area via multiple methods including Internet advertisements, posted flyers, and referral through local emergency departments. The presence of mTBI was defined in agreement with the American Congress of Rehabilitation Medicine guidelines including (1) alteration of mental status related to specific head trauma lasting up to 24 h; (2) loss of consciousness <30 min; (3) post-traumatic amnesia lasting <1 day; and (4) initial Glasgow Coma Scale between 13 and 15 (61). To be eligible, participants were required to provide written documentation from a medical provider or other professional who either witnessed the injury or provided immediate treatment or care as a result of the injury.

All participants were right-handed and reported English as their first language. Individuals were not eligible to participate in this study in the presence of (1) any contraindications for MRI, (2) education <9th grade, (3) history of alcoholism or substance abuse, (4) colorblindness, or (5) lifetime history of DSM-IV Axis I disorder. Healthy control participants were additionally ineligible with any lifetime history of TBI or sport participation in concussion high-risk sports (e.g., football, rugby, boxing, ice hockey, wrestling, soccer, or martial arts) for longer than 1 month. All participants were compensated for their time. All study procedures were approved by The University of Arizona Institutional Review Board and the US Army Human Research Protections Office. All participants provided written informed consent prior to participation. Participant demographics are further summarized in Table 1.

**Table 1:** Participant demographics and self-report measures.

	**Healthy Control**	**mTBI**	**Statistic^a^**	***p***	**Effect Size^b^**
	**Mean (*SD*)**	**Mean (*SD*)**			
***n***	18	34			
**DEMOGRAPHICS**					
Age (years)	23.2 (3.4)	24.4 (7.4)	−0.795	0.430	−0.232
Height (in)	67.2 (4.3)	66.4 (4.2)	0.649	0.520	0.189
Weight (lb)	158.4 (41.5)	151.8 (38.1)	0.565	0.576	0.165
Total mTBIs^c^	0 [0]	2 [1]	−12.6	<0.001	−3.673
Sex (n)			0.272^f^	0.602	0.144
Male	9	13			
Female	9	21			
Race/Ethnicity (n)			4.063^d^	0.540	0.577
Asian/Pacific Islander	2	3			
Black/African American	0	2			
Hispanic/Latino	1	0			
Native American/ American Indian	2	2			
Other	0	1			
White	12	25			
Weeks Post-Injury (n)					
2 weeks		6 (17.6%)			
4 weeks		8 (23.5%)			
12 weeks		7 (20.6%)			
24 weeks		6 (14.7%)			
52 weeks		9 (23.5%)			
Mechanism of Injury (n)					
Sports-related		13 (38.2%)			
Slip and/or fall		7 (20.6%)			
MVA		6 (17.6%)			
Bike related		4 (11.8%)			
Environmental^e^		3 (8.8%)			
Assault		1 (2.9%)			
**SELF-REPORT MEASURES**					
PSQI Total Score	3.7 (1.8)	6.8 (3.5)	−4.227	<0.001	−1.232
BDI–II Total Score	2.4 (2.9)	9.6 (8.1)	−4.636	<0.001	−1.351
RPQ-3	0.2 (0.6)	2.4 (2.5)	−4.771	<0.001	−1.391
RPQ-13	0.3 (1.0)	10.7 (10.6)	−5.616	<0.001	−1.637
SWLS Total Score	26.6 (6.0)	26.2 (4.9)	0.230	0.819	0.067
GOS-E Outcome^f^ (n)					
Upper Good Recovery		10			
Lower Good Recovery		13			
Upper Moderate Disability		10			
Upper Severe Disability		1			

a *Tests are two-tailed t-tests unless otherwise indicated*.

b *Cohen's d effect sizes*.

c *Data presented as median [interquartile range]*.

d *χ^2^ test*.

e *Mechanism of injury is for the most recent mTBI. Environmental accidents include falls from ladders or unanticipated contact with environmental features (ground, structures) unrelated to sports or falls*.

f *No GOS-E data were collected on the healthy control participants*.

*mTBI, mild traumatic brain injury; MVA, motor vehicle accident; PSQI, Pittsburgh Sleep Quality Index; BDI-II, Beck Depression Inventory – 2; RPQ, Rivermead Post-concussion Symptom Questionnaire; SWLS, Satisfaction with Life Survey; GOS-E, Glasgow Outcome Scale-Extended*.

### Materials and procedure

As part of a larger, on-going study, individuals in the current sample were evaluated at one of six pre-specified time points relative to their injury date: 2-weeks, 4-weeks, 3-months, 6-months, or 12-months post-injury (see Table 1). The purpose of the larger study is to examine structural and neural correlates of neuropsychological, behavioral, and self-reported outcomes over the first year following an mTBI. Individuals are included at only one of six time points and treated as exemplars of recovery at that time. We report here on a subset of the outcomes.

All participants underwent a comprehensive neuropsychological evaluation, including several self-report measures (described below), followed by a neuroimaging session that included diffusion-weighted imaging (DWI). Only a subset of the outcome measures are presented here. In addition to indices of depressive symptoms and sleep quality, we included NINDS CDEs related to global outcomes, post-mTBI symptom presentation, and perceived health-related quality of life, all of which may be impacted by depression and/or lower sleep quality.

### Self-report outcomes

#### Glasgow outcome scale - extended (GOS-E)

The GOS-E is a structured interview commonly used to assess overall disability and recovery following TBI (62). It is a core element of the NINDS CDEs for all levels of severity of TBI, including sport-related concussion. Scores on subscales for the GOS-E quantify disability in cognition, independence, employability, and social or community participation. These subscales are cumulatively reported as a single overall outcome, ranging from 1 (death) to 8 (upper good recovery). Reliability for the GOS-E is high [κ = 0.85; (62)].

#### Beck depression inventory-II (BDI-II)

The BDI-II is a self-administered survey requiring self-appraisal of mood over the preceding 2 weeks (63, 64). Increasing scores on the BDI-II are associated with increasing levels of depression symptoms. The BDI-II has high test-retest reliability (*r* > 0.9), as well as construct (vs. the original BDI) and moderate concurrent (vs. the State-Trait Anxiety Inventory Anxiety and Depression factors) validity (*r* > 0.68; (65, 66)). Previous work has demonstrated that individuals with both recent mTBIs and a history of mTBI report higher levels of depression and increased likelihood for depression by comparison to individuals without mTBI (22, 67–71).

#### Pittsburgh sleep quality index (PSQI)

The PSQI is an 18-item self-report questionnaire yielding information about overall sleep quality, latency, duration, efficiency, disturbances, medication use, and the effects on daytime function (60). Better sleep quality is associated with lower scores, with scores of >5 indicating poor sleep and >8 indicating insomnia. Prior work has indicated good test-retest reliability (*r* > 0.80; (60)) and sensitivity to sleep disruption following mTBI (72, 73).

#### Satisfaction with life survey (SWLS)

The SWLS is a self-administered survey in which individuals assess current life satisfaction based on five questions. Questions are scored “Strongly Disagree” (1) to “Strongly Agree” (7) and summed with a maximum value of 35 (74). Higher scores indicate greater life satisfaction. The SWLS has demonstrated test-retest reliability [*r* > 0.80; (75)]. It is a basic element of the NINDS CDEs for concussion and mTBI (59). Previous research has indicated that individuals with prior mTBIs report greater life dissatisfaction (76, 77).

#### Rivermead post-concussion symptom questionnaire (RPCSQ)

The RPCSQ is a common post-mTBI assessment of symptom presentation and is a basic element of the NINDS CDEs for concussion and mTBI (59). Participants self-report the extent to which 16 symptoms currently affect them compared to preinjury-levels. Ratings range from “Not experienced at all” (0) and “No more of a problem” (1) to “A severe problem (4) (78). Previous analyses of the RPCSQ have identified a two-factor structure, such that the first three questions (RPQ3) are sensitive to acute injury symptoms while the final 13 (RPQ13) are sensitive to chronic symptoms. These two scales have good test-retest reliability (*r* > 0.70) and external validity [ρ > 0.60; (79)], and the RPQ is a significant predictor of 3-month outcomes (80).

### Diffusion-weighted imaging

We acquired DWI data using single-shot echo planar imaging (EPI) (TE = 88 ms; TR = 9600 ms; acquisition matrix = 128 × 128; FOV: 256 × 256; slice thickness = 2 mm with no gap) on a Siemens Skyra 3.0 Tesla MRI machine (32-channel head coil; MAGNETO Skyra Siemens Healthcare). Images were acquired following a within-lab standardized process, including cross checks on image acquisition parameters at the time of scanning, with a single MRI technician overseeing all scanning procedures. Diffusion gradients were applied along 72 directions, with b = 1000 s/mm^2^ and six non-diffusion weighted images (b_0_). Preprocessing followed the standard pipeline available through FMRIB Software Library's [FSL; (81)], including EPI distortion correction using *TOPUP* (82), motion and eddy current distortion correction using *eddy* (83), skull-stripping with the brain extraction tool [*BET*; (84)], and diffusion tensor model fitting using *DTIFIT* (85). Output from *DTIFIT* includes separate images for FA, MD and three eigenvalues (λ_1_, λ_2_, λ_3_). λ_1_ is the axial diffusivity and radial diffusivity is the average of λ_2_ and λ_3_.

The four DTI-metric images (FA, MD, AD, and RD) were then nonlinearly registered to a standard template (FMRIB-58) followed by affine-alignment to standard space (MNI, 1 × 1 × 1 mm) using Tract-Based Spatial Statistics [TBSS; (86)]. A study-specific, averaged whole-brain skeletonized FA mask was created through TBSS (threshold = 0.2). Skeletonizing in this way reduces the number of voxels considered in statistical modeling by only including voxels near the center of white matter tracts.

Whole-brain voxel-wise statistical analysis was conducted via FSL's *randomise* using threshold-free cluster enhancement (87) with 5000 permutations. Significant voxels were identified as those with *p* < 0.05 after family-wise error rate (FWER) adjustment for multiple comparison. We tested between-group differences using a two-sample *T-*test for each of the DTI metrics (FA, MD, AD, RD), controlling for age, sex, and days post-injury. For the self-report measures, we fit within-group GLMs for each combination of measure and metric, controlling for age, sex, and days since injury (mTBI only). Within-group correlations for the relationships between DTI metrics and self-report outcome measures were examined.

A mask of all significant voxels was created for each of the DTI metrics. These masks were then used to compute the mean DTI-metric value for each participant, which was then extracted for *post-hoc* analyses. Mean DTI-metric values and self-report measures were scatter-plotted and the partial correlation coefficient, after controlling for age, sex, and days post-injury, was computed. Significant white matter voxels were anatomically identified using the Johns Hopkins University (JHU) ICBM-DTI-81 White-Matter Labels atlas (88).

### Statistical analyses

Between-group comparisons for continuous demographic characteristics and self-report measures were analyzed using a two-tailed *T*-test in R [v. 3.4.2; (89)]. The between-group gender and ethnicity comparisons were computed using a Chi-square test. Average values over all significant voxels were plotted in R as scatterplots with the relevant self-report outcomes using ggplot2 (90). For the healthy control participants, taking an inverse transformation of the BDI scores [$y=\frac{1}{(x+1}$)] and a square transformation (*y* = *x*^2^) of the SWLS scores was necessary to reduce skewness. No GOS-E data were collected for the healthy control participants, as this measure is specific to brain injury.

## Results

### Demographic and self-report measures

Demographic characteristics and self-report outcomes are summarized in Table 1. No participants were active duty military; however, we did not query for Veteran status. The groups did not differ in age, height, weight, or gender. Brain injured participants reported a median number of mTBIs, including the one used for referral, of 2 (range: 1–4). Most individuals (*n* = 13) reported a sports-related mechanism of injury for the referring mTBI. The mTBI participants reported significantly more post-concussive symptoms, poorer sleep quality, and greater depressive symptoms than the healthy control participants. The majority of the mTBI participants (*n* = 23, 68%) reported a good recovery as assessed via the GOS-E.

Given the dynamic nature of post-mTBI recovery and the role that time since injury may play, we additionally report the means and standard deviations for the self-reported outcomes for each of the six distinct time points in Table 2. Given the small sample sizes across all of the post-mTBI groups, we do not report any between-group statistical comparisons at this time.

**Table 2:** Participant demographics and self-report measures by weeks post-Mtbi.

	**Uninjured**	**2 weeks**	**4 weeks**	**3 months**	**6 months**	**1 year**
***n***	***n* = 18**	***n* = 6**	***n* = 8**	***n* = 7**	***n* = 5**	***n* = 8**
**DEMOGRAPHICS**						
Age (years)	23.2 (3.4)	25.1 (10.1)	25.3 (6.7)	26.6 (9.3)	24.9 (8.6)	20.9 (1.4)
Height (in)	67.2 (4.3)	69.3 (6.1)	66.9 (3.5)	65.3 (3.9)	65.2 (2.6)	65.5 (4.3)
Weight (lb)	158.4 (41.5)	169.7 (43.9)	158.9 (36.5)	139.3 (34.6)	157.0 (49.2)	139.0 (32.1)
Total mTBIs^a^	0.0 [0.0]	2.0 [0.8]	2.0 [1.0]	2.0 [2.0]	2.0 [2.0]	2.0 [1.0]
**Sex (*****n*****)**						
Male	9 (50%)	4 (66.7%)	4 (50%)	2 (28.6%)	1 (20%)	2 (25%)
Race/Ethnicity (n)						
Asian/Pacific Islander	2	0	0	1	0	2
Black/African American	0	2	0	0	0	0
Hispanic/Latino	1	0	0	0	0	0
Native American/ American Indian	2	0	1	0	1	0
Other	0	0	1	0	0	0
White	12	4	5	6	4	6
**SELF-REPORT MEASURES**						
PSQI Total Score	3.7 (1.8)	6.7 (4.5)	7.0 (1.4)	6.0 (2.8)	7.2 (3.8)	7.1 (5.0)
BDI-II Total Score	2.1 (2.6)	9.3 (7.2)	9.8 (6.5)	12.6 (10.3)	9.2 (9.8)	5.1 (4.4)
RPQ-3	0.2 (0.6)	2.7 (3.7)	3.8 (2.3)	2.0 (2.3)	1.4 (1.9)	1.9 (2.2)
RPQ-13	0.3 (1.0)	11.7 (9.3)	12.4 (10.0)	9.0 (11.0)	13.2 (16.4)	8.1 (9.8)
SWLS Total Score	26.6 (6.0)	28.2 (2.8)	24.1 (6.2)	25.0 (6.5)	25.6 (4.4)	28.1 (2.8)
GOS-E Outcome^b^ (*n*)						
Upper Good Recovery		2	2	3	1	2
Lower Good Recovery		2	1	2	3	5
Upper Moderate Disability		1	5	2	1	1
Upper Severe Disability		1	0	0	0	0

a *Data presented as median [interquartile range]*.

b *No GOS-E data were collected on the healthy control participants. mTBI, mild traumatic brain injury; PSQI, Pittsburgh Sleep Quality Index; BDI-II Beck Depression Inventory – 2; RPQ, Rivermead Post-concussion Symptom Questionnaire; SWLS, Satisfaction with Life Survey; GOS-E, Glasgow Outcome Scale – Extended*.

### Diffusion metrics

#### Whole-brain group differences

After correcting for multiple comparisons, there were no statistically significant differences observed between the healthy control participants and those with a history of mTBI for any of the diffusion metrics at the whole brain level (*a priori* α = 0.05). However, there were voxels with a trend toward greater radial diffusivity in the mTBI participants than the healthy control participants (FWER 0.064 ≤ *p* ≤ 0.094; Supplementary Figures 1, **5**, top row).

#### Correlation between diffusion metrics and self-report measures

**Figure 1:**
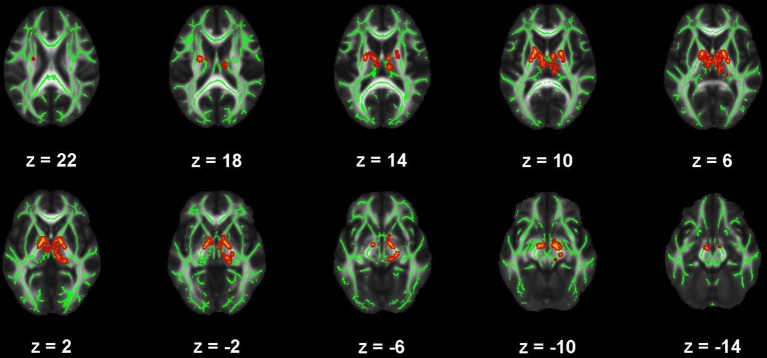
Map of voxels with significant correlations between fractional anisotropy (FA) and Beck Depression Inventory – II (BDI) total scores in the mild traumatic brain injury (mTBI) participants. The average white-matter skeleton is presented in green. Yellow voxels indicate significant, negative correlations between FA and BDI total score (family-wise error rate corrected *p* < 0.05). Surrounding voxels are filled in red for visual purposes only. Images are in neurological orientation and Z-coordinates are presented in MNI standard space.

**Figure 2:**
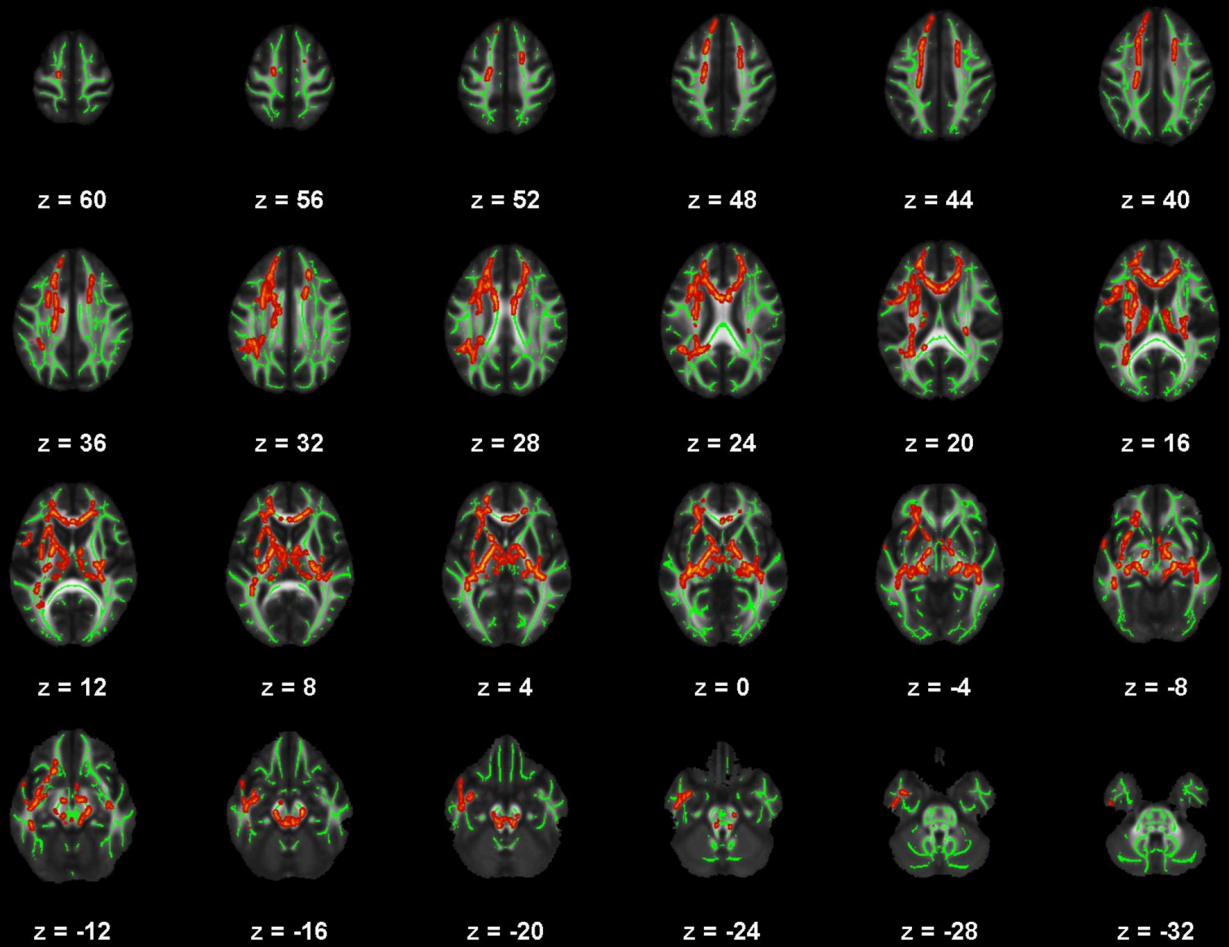
Map of voxels with significant correlations between radial diffusivity (RD) and Beck Depression Inventory – II (BDI) total scores in the mild traumatic brain injury (mTBI) participants. The average white-matter skeleton is presented in green. Yellow voxels indicate significant, positive correlations between RD and BDI total score (family-wise error rate corrected *p* < 0.05). Surrounding voxels are filled in red for visual purposes only. Images are in neurological orientation and Z-coordinates are presented in MNI standard space.

**Figure 3:**
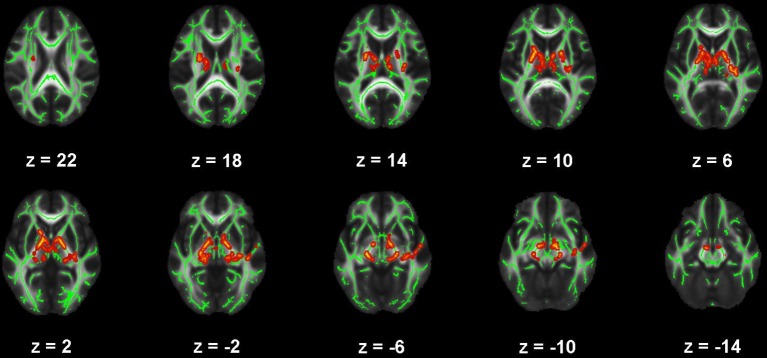
Map of voxels with significant correlations between mean diffusivity (MD) and Beck Depression Inventory – II (BDI) total scores in the mild traumatic brain injury (mTBI) participants. The average white-matter skeleton is presented in green. Yellow voxels indicate significant, positive correlations between MD and BDI total score (family-wise error rate corrected *p* < 0.05). Surrounding voxels are filled in red for visual purposes only. Images are in neurological orientation and Z-coordinates are presented in MNI standard space.

**Figure 4:**
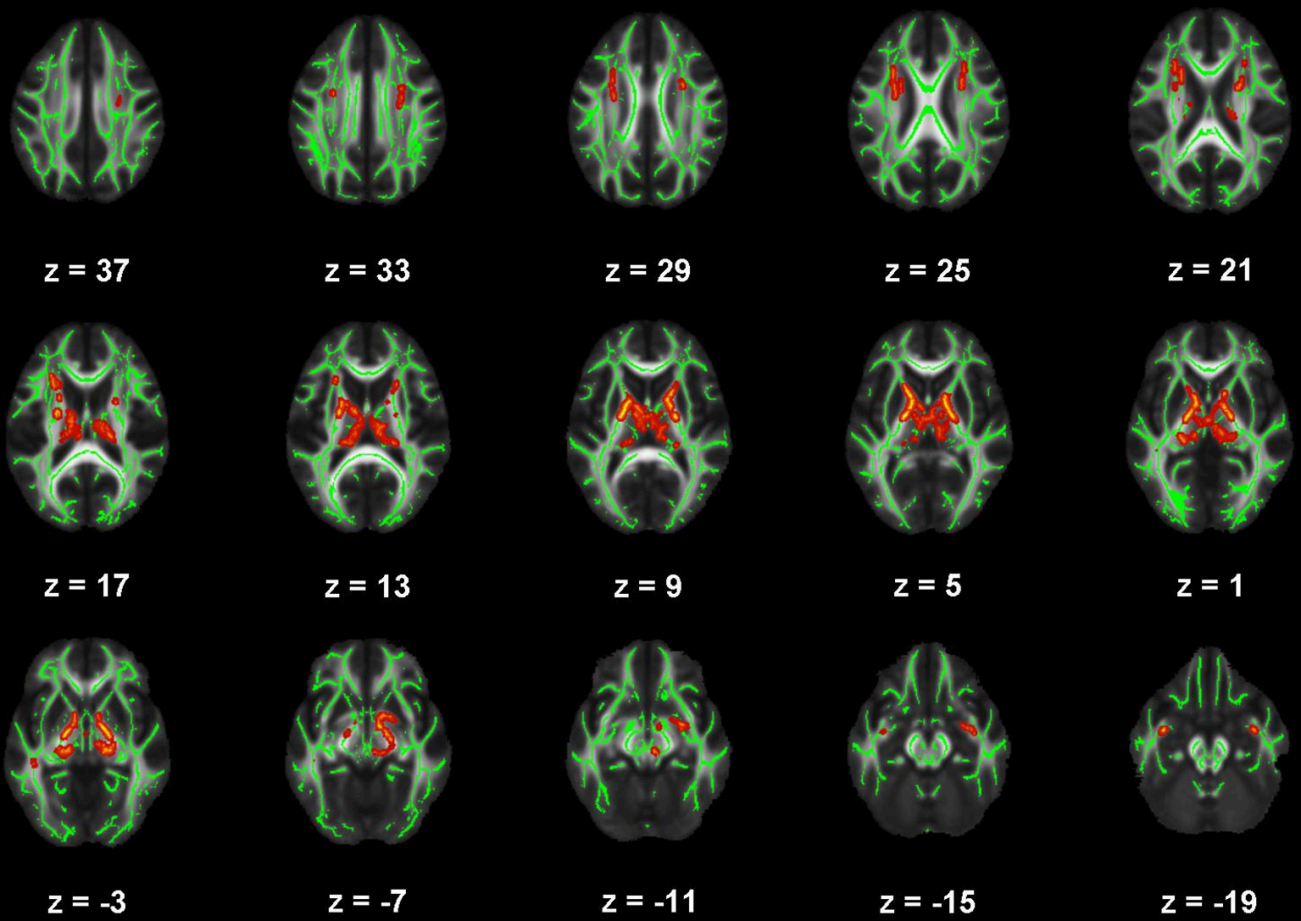
Map of voxels with significant correlations between fractional anisotropy (FA) and Pittsburgh Sleep Quality Index (PSQI) total scores in the mild traumatic brain injury (mTBI) participants. The average white-matter skeleton is presented in green. Yellow voxels indicate significant, negative correlations between FA and PSQI total score (family-wise error rate corrected *p* < 0.05). Surrounding voxels are filled in red voxels for visual purposes only. Images are in neurological orientation and Z-coordinates are presented in MNI standard space.

**Figure 5:**
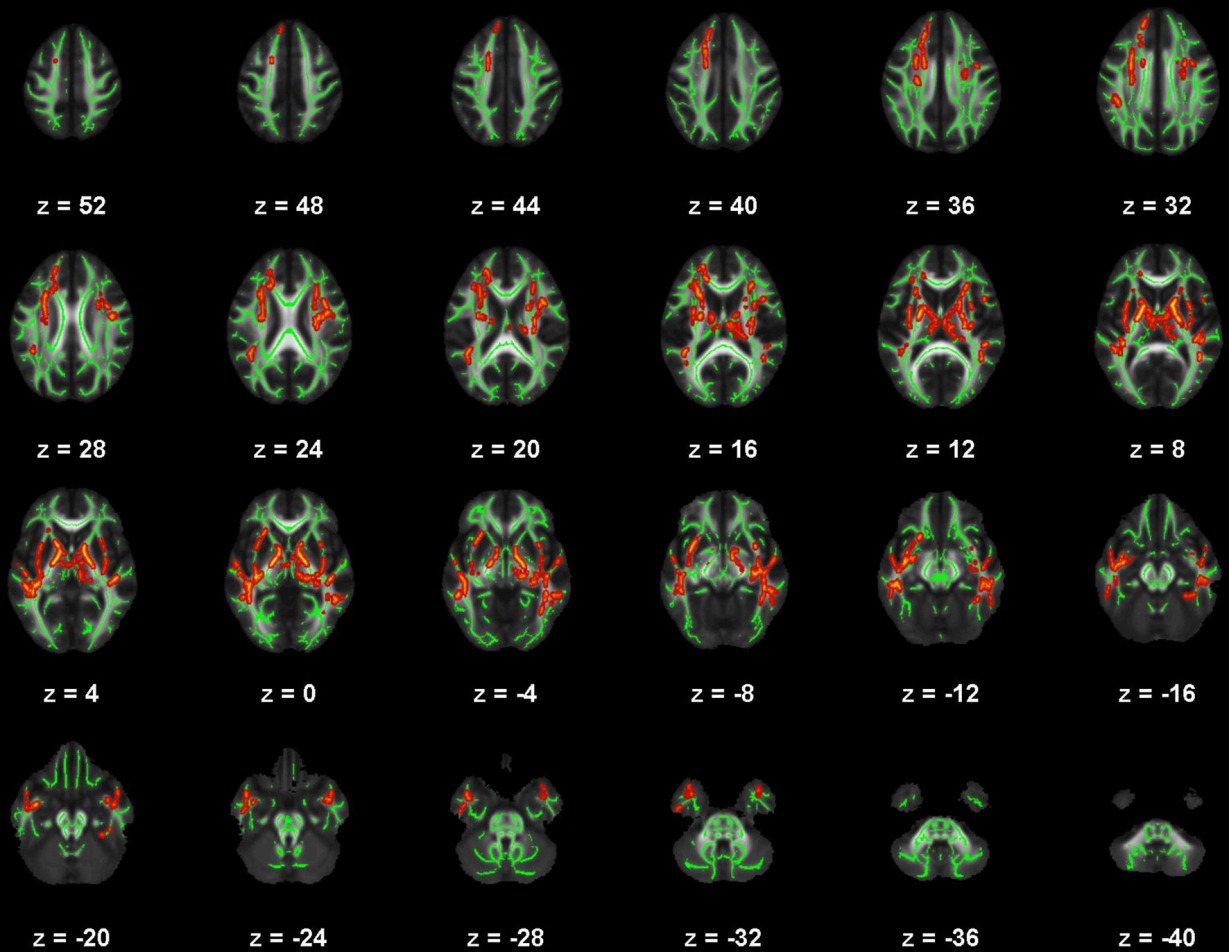
Map of voxels with significant correlations between radial diffusivity (RD) and Pittsburgh Sleep Quality Index (PSQI) total scores in the mild traumatic brain injury (mTBI) participants. The average white-matter skeleton is presented in green. Yellow voxels indicate significant, positive correlations between RD and PSQI total score (family-wise error rate corrected *p* < 0.05). Surrounding voxels are filled in red for visual purposes only. Images are in neurological orientation and Z-coordinates are presented in MNI standard space.

**Figure 6:**
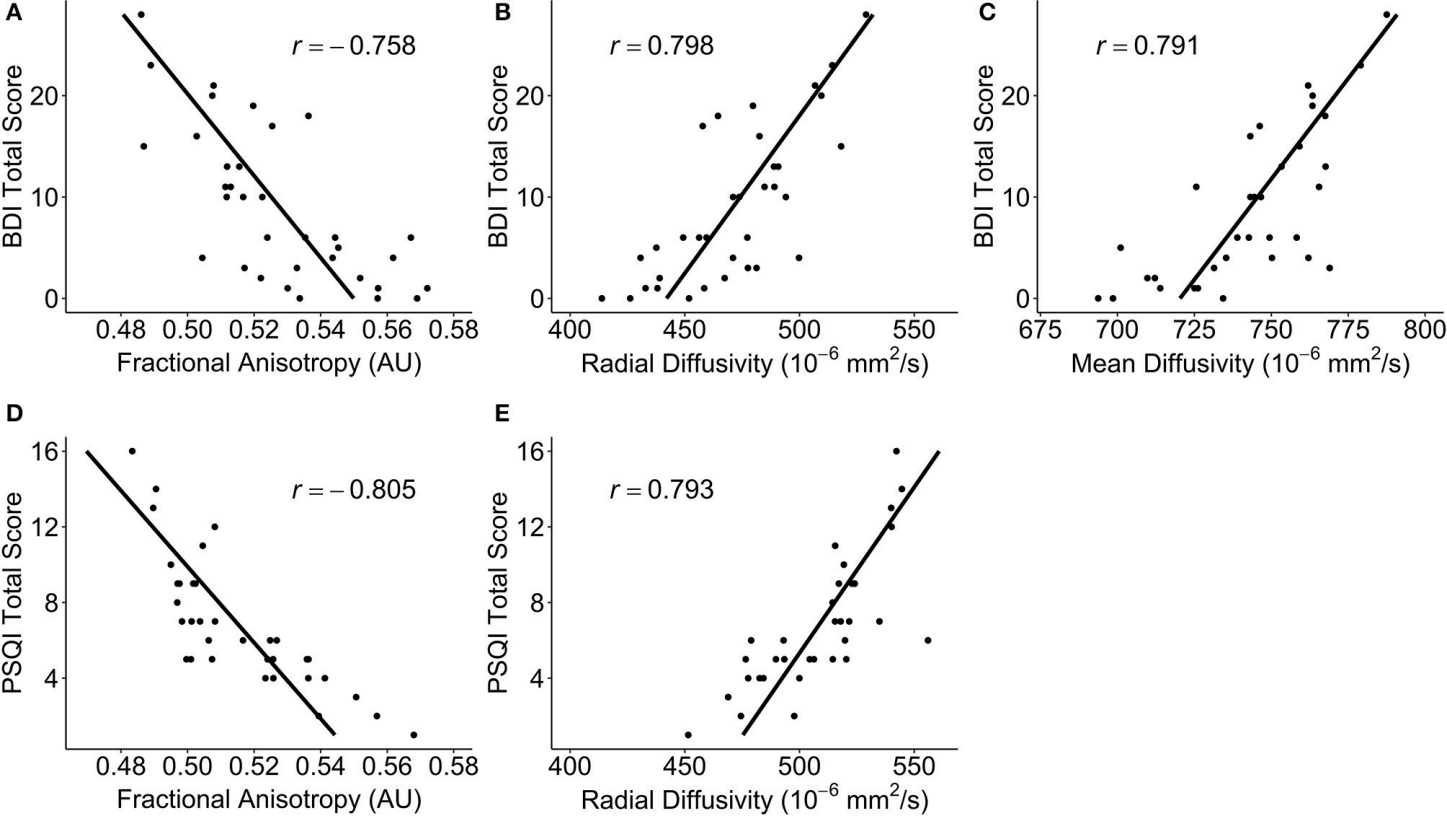
Scatterplots of *post-hoc* partial correlations between diffusivity measures and self-reported outcomes for participants with a mild traumatic brain injury. The points in each plot are the average DTI measure over all of the significant voxels. The black line is a regression line predicting the self-report outcomes from the diffusivity measures, controlling for age, sex, and days post-injury. Correlation coefficients are noted as *r*. **(A)** The Beck Depression Inventory – II (BDI) total score vs. fractional anisotropy (FA); (**B)** BDI total score vs. radial diffusivity (RD); **(C)** BDI total score vs. mean diffusivity (MD); **(D)** Pittsburgh Sleep Quality Index (PSQI) total score vs. FA; **(E)** PSQI total score vs. RD.

There were no significant correlations within the healthy control group between any of the DTI metrics and any of the self-report outcomes. However, a positive trend association between AD and SWLS scores was observed (0.068 ≤ *corrected p* ≤ 0.099, Supplementary Figures 2, **5**, second row). We found voxels with significant correlations in the mTBI participant group for the BDI-FA (*corrected p* < 0.05, Figures 1, 6A), BDI-MD (*corrected p* < 0.05, Figures 2, 6B), BDI-RD (*corrected p* < 0.05, Figures 3, 6C), PSQI-FA (*corrected p* < 0.05, Figures 4, 6D) and PSQI-RD (*corrected p* < 0.05, Figures 5, 6E) relationships. Finally, trend associations were observed for RPQ3-AD (0.093 ≤ *corrected p* ≤ 0.1, Supplementary Figures 3, **5**, third row) and PSQI-MD (0.079 ≤ *corrected p* ≤ 0.1, Supplementary Figures 4, 5, bottom row). No statistically significant correlations were present in the mTBI group between any diffusion metric and the GOS-E, RPQ13, or SWLS scores.

**Figure 7:**
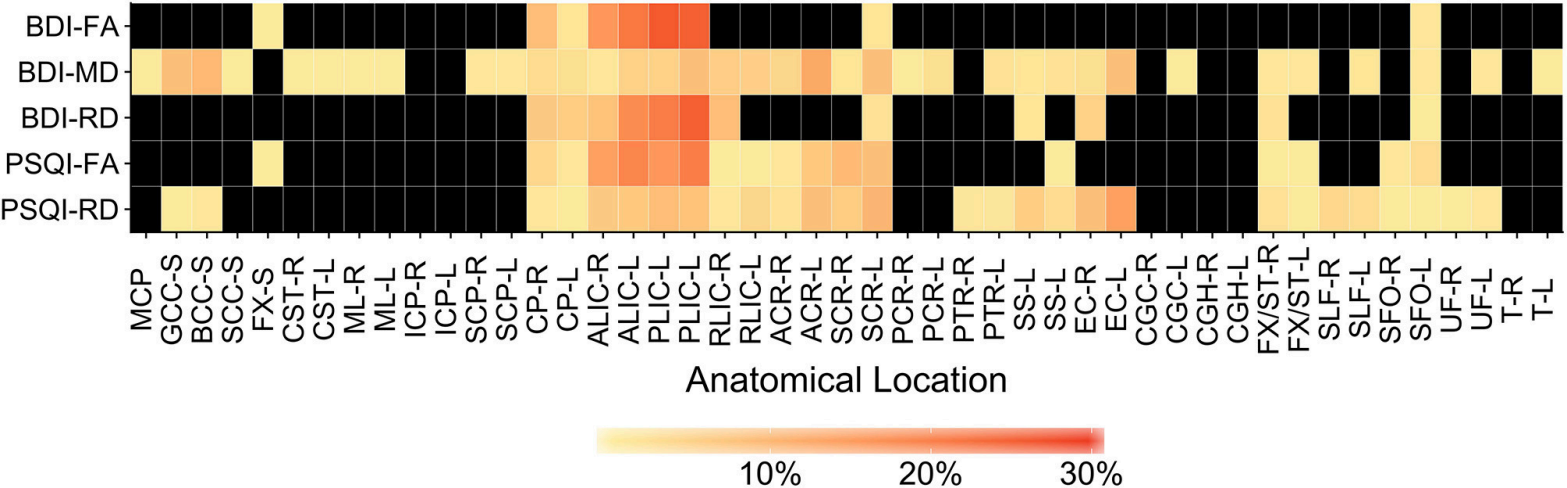
Heatmap showing distribution of labeled, significantly correlated voxels (family-wise error rate corrected *p* < 0.05) for each correlation pair. Anatomical labels are drawn from the JHU ICBM-DTI-81 White-Matter Labels atlas and retrieved using the FMRIB Software Library (FSL) *atlasquery* function. *Atlasquery* returns the probability (and, in the case of the JHU ICBM-DTI-81 atlas, the proportion) of voxels in a mask belonging to a region identified in a given atlas. The JHU ICBM-DTI-81 atlas does not encompass all white matter, and consequently some voxels remain unclassified. Colors reflect the percentage of labeled voxels identified within each anatomical location ($\frac{localized\;voxels}{\sum \left(classified\;voxels\right)}$. Black boxes indicate no voxels with a significant correlation were present in that anatomical location. BDI, Beck Depression Inventory; PSQI, Pittsburgh Sleep Quality Index; FA, Fractional Anisotropy; MD, Mean Diffusivity; RD, Radial Diffusivity. Anatomical location abbreviations are summarized in Supplementary Table 1.

Anatomical locations of significant correlations were automatically determined using FSL's *atlasquery* function and the JHU ICBM-DTI-81 White-Matter Labels atlas (88). These locations are summarized in Figure 7 and the abbreviations detailed in Supplementary Table 1. *Atlasquery* returns the probability (and, in the case of the JHU ICBM-DTI-81 atlas, the proportion) of voxels in a mask belonging to a region identified in a given atlas. The JHU ICBM-DTI-81 atlas does not encompass all white matter, and consequently some voxels remain unclassified. Figure 7 has been rescaled to reflect only the classified voxels (e.g., 25% = 25% of the classified voxels). Across all five of the significant correlation pairs (BDI-FA, BDI-MD, BDI-RD, PSQI-FA, and PSQI-RD), correlations were consistently observed bilaterally within the internal capsules (anterior and posterior limbs), corona radiata (anterior and superior), fornix, and superior fronto-occipital fasciculi.

#### Confirmatory *post-hoc* analyses

We performed two additional *post-hoc* analyses to further strengthen these findings. To confirm the lack of PSQI-DTI and BDI-DTI correlation in the healthy controls, we conducted an ROI analysis. Using the significant voxel masks created for the mTBI participants, the healthy control participants' mean DTI-metric values were extracted in the same manner as for the mTBI participants. *Post-hoc* partial correlations for the healthy controls were computed as described above, controlling for age and sex. None of these *post-hoc* correlations were statistically significant, as anticipated based on the results from *randomise*. Additionally, we compared the partial correlation coefficients between the healthy control and mTBI participants (91). These analyses confirmed that the observed correlations in the mTBI participants were significantly different from those in the healthy controls (see Supplementary Table 2).

Additionally, the BDI-II includes two questions that specifically address sleep. We observed significant correlations between sleep quality (PSQI) and DTI metrics, as well as depressive symptoms (BDI) and the same DTI metrics (FA and RD). Additionally, there was overlap in the structural areas exhibiting significance. Consequently, it was important to examine whether the BDI-DTI correlations for the mTBI participants depended upon perceived sleep quality. We computed a modified BDI total score that ignored items 16 (changes in sleeping pattern) and 20 (tiredness or fatigue) and re-calculated the partial correlations between BDI and the mean FA, MD, and RD over the previously identified significant voxels, while controlling for age, sex and time since injury. We then compared the two sets of correlation coefficients (91). There were no significant differences between the original and adjusted BDI correlations (see Supplementary Table 2), suggesting that the BDI-FA, BDI-MD, and BDI-RD correlations were not necessarily dependent on self-reported sleep characteristics.

## Discussion

The focus of this study was to identify neural correlates of clinically-relevant self-report outcomes related to global outcomes, psychiatric and psychological status, perceived health-related quality of life, and post-concussive related symptoms (59) as well as self-reported sleep quality (60). Specifically, the emphasis here was on metrics related to white-matter integrity, including FA as well as MD, AD, and RD. We hypothesized that individuals with a recent mTBI would exhibit lower FA and greater MD and RD than individuals with no prior history of mTBI. Additionally, we hypothesized that within-group correlations would be present such that poorer outcomes (e.g., poorer sleep quality, more depression symptoms) would be associated with lower FA and greater MD and RD. These hypotheses were partially confirmed.

### DTI sensitivity to mTBI

Contrary to our hypotheses, we did not observe any *statistically significant* differences between the healthy control and mTBI participants for any of the four diffusion metrics. This lack of difference occurred despite participants with mTBI reporting statistically greater sleep disturbances, depression symptoms, and post-concussive symptoms than the healthy controls. Recent systematic reviews and meta-analyses have generally highlighted the sensitivity of FA and MD to mTBI (40, 41, 53), however, the directional difference relative to non-mTBI participants is unclear, with lower, higher, and no differences reported (31, 43, 46, 48, 50–52). Our findings here are consistent with those of “no differences” at a whole brain corrected level, however it is important to note that generalization across DTI studies in mTBI is limited by cross-study heterogeneity in sample sizes, participant ages and populations, imaging protocols, and processing methods (40, 41). Please see **Exploratory trends** for a further discussion of group differences.

### Correlations with self-report outcomes

Consistent with our hypotheses, both sleep quality and depressive symptoms in the participants with mTBIs in the present study were correlated with DTI metrics. The relationships with depressive symptoms remained significant after removing sleep-related items from the BDI total score, suggesting that these were not driven by sleep issues *per se*. These measures exhibited negative correlations with FA and positive correlations with RD in projection and association tracts, including the internal capsule (IC), superior and anterior corona radiata (SCR, ACR), anterior and posterior thalamic radiations (ATR, PTR), and superior fronto-occipital fasciculus (SFO). Collectively, these white-matter tracts are integral aspects of neural circuits connecting deep brain structures, specifically the thalamus, parietal, and occipital cortical regions with frontal and prefrontal cortex areas. These connections not only play a critical role in sleep-wake regulation (thalamo-cortical circuits; (92–94), but also facilitate information processing, cognitive control, attention, executive function, and emotion regulation (95–97), all of which may be impaired following mTBI. Recent models of the neural basis of depression have further illustrated how alterations in the information processing supported by these prefrontal-posterior cortical/subcortical pathways (e.g., schema-guided attention, interpretation, and cognitive control processes) may bi-directionally interact with sleep quality to produce/maintain depressive symptoms (98). Consequently, damage in these pathways may precipitate the clinical presentation of mTBI, especially with respect to the correlated sleep and depression-related symptoms we observed in our sample.

There is substantial evidence that prior mTBI is associated with poor sleep quality, both self-reported (8, 12, 99, 100) as well as when measured via actigraphy (11, 101) and/or polysomnography (102). Poor sleep quality may manifest in numerous ways, including insomnia (8, 12), hypersomnia (10), pleiosomnia (11), and increased night-to-night sleep variability (101). Proposed mechanisms for sleep-wake disturbances following mTBI include reduced sleep-wake regulation neurotransmitter availability, specifically low hypocretin/orexin, and lower counts of wake-promoting neurons in the hypothalamus (103–105). To our knowledge, the findings here are the first to link sleep quality with white-matter integrity following mTBI, and suggest that there are likely overlapping relationships between these mechanisms.

The results reported here are consistent with findings from both depression- and sleep-related studies apart from mTBI [i.e., where sleep disturbance is thought to both promote, and be promoted by, underlying neural processing abnormalities in depression; (98)]. Lower FA in the SLF, IC, and corpus callosum are frequently observed in major depressive disorder (54). Additionally, individuals with poor sleep quality (57), increased sleep variability (58), and insomnia (55, 56) all exhibit lower FA, particularly in the IC, SLF, and thalamic radiations. Importantly, previous work has indicated that poor sleep quality is associated with lower FA and increased RD even in healthy individuals and can cause reduced myelination and limit oligodendrocyte precursor proliferation (57, 58). In light of mouse models indicating that mTBI can directly result in loss of myelination (35–38), it is unclear to what extent post-mTBI sleep quality leads to white-matter damage vs. trauma-induced white-matter damage leading to poor sleep. Regardless, white-matter damage in these pathways may explain overlapping presentations of poor sleep quality, psychological distress, and cognitive impairment typically associated with mTBI. Identifying the independent contributions of traumatic insult vs. sleep loss-induced alterations in white-matter remains an open area of investigation in this population.

### Exploratory trends

Surprisingly, and generally contrary to the bulk of the literature on mTBI and DTI, there were no statistically significant differences (at a whole brain FWER *corrected p* < 0.05 level) between the mTBI participants and healthy controls. We did, however, observe a trend toward greater RD in the mTBI participants (at a FWER *corrected p* < 0.1), primarily in the right hemisphere. While areas did not overlap exactly with the significant voxels from the mTBI group correlations, they do exist within the same pathways, particularly the corona radiata, longitudinal fasciculi, and the corpus callosum. While these differences do not meet the conventional level of significance, they do point to the possibility of myelin-related damage following mTBI.

Similarly, despite the observed relationships between FA/RD and both sleep quality and depressive symptoms, there were no statistically significant correlations (at a FWER *corrected p* < 0.05 level) between diffusion measures and post-concussive symptom presentation on the Rivermead Post-concussion Symptom Questionnaire. However, a negative *trend* did exist between AD and post-concussive symptoms on the RPQ3 (which identifies somatic symptoms; i.e., headaches, dizziness, nausea), such that lower AD was associated with greater symptom presentation. This result is in line with other recent findings (106), suggesting that somatic symptom presentation may be related to axonal damage.

### Limitations

The present study indicates an association between diffusion metrics and self-report outcomes subsequent to mTBI, particularly with regard to sleep and depressive symptoms. However, a number of challenges remain. First, our overall sample was relatively small, which may contribute to the lack of statistically significant differences observed between healthy control participants and mTBI participants. Secondly, despite significant correlations between diffusion metrics and both sleep quality and depressive symptoms, the findings here present only a cross-sectional view of post-mTBI outcomes. Consequently, we cannot make any assertions about causation with respect to either the white matter integrity or self-report outcomes.

Third, our group had considerable heterogeneity of time since injury, ranging from 2-weeks to 12-months post injury. There is reasonable evidence that diffusion-related metrics may change over the weeks to months following an mTBI (40). Given the exclusively cross-sectional nature of our data, we addressed this potential limitation in the following ways. First, both our between-group and within-group models controlled for days since injury. Second, a *post-hoc* mTBI participant within-group correlation between the mean significant voxel values for PSQI and BDI-II scores reported earlier and time since injury revealed a non-significant correlation (*r* = 0.026, *p* = 0.146). Therefore, while intra-individual DTI-metrics may typically change over the course of mTBI recovery (40), the relationships between DTI measures, sleep quality, and depression we observed in the present sample appear independent of time since injury.

Finally, the white matter skeleton created during TBSS is based upon FA local maxima, generally near the midline of the white-matter tract (107). Thus, group differences between controls and mTBI participants may be present in non-maxima areas of the tracts, but these potential differences would not be detectable using the methods employed here. Finally, there are no established cutoffs, or reliable change indices, for DTI metrics after mTBI to identify whether the observed relationships reflect clinically meaningful changes in diffusion. Future work should address longitudinal outcomes, ideally with pre-injury DTI (though we recognize the inherent challenge in that), as well as machine-learning-based modeling methods (e.g., cross-validated logistic regression, classification trees) to identify discriminative post-mTBI changes in DTI metrics.

## Conclusion

The results of this study contribute to a growing body of literature indicating that there are correlations between white-matter structure and clinical measures related to sleep quality and depression following mTBI. We have identified that the self-reported presentation of poor sleep quality and depressive symptoms following mTBI correlates with lower white-matter integrity in multiple areas of the brain involved in sleep-wake cycle and emotion regulation, in addition to information processing, cognitive control, attention, and executive function. Finally, trends in our data suggest that there may be alterations in white-matter structure that distinguish individuals with a history of mTBI from those without. Future work should emphasize identifying cutoff values in DTI metrics that provide clinically meaningful distinctions between individuals. Such findings will help not only to continue to increase what is known about mTBI pathophysiology and recovery, but will also help to guide best practices for the diagnosis and treatment of mTBI.

## Author contributions

AR conducted the MRI data processing, statistical analyses, and drafted the initial manuscript. SB, ND, RS, and AA assisted with data interpretation and manuscript revisions. BS assisted with manuscript revisions. WK designed the study, assisted with data interpretation and critique, as well as manuscript review and revisions.

### Conflict of interest statement

The authors declare that the research was conducted in the absence of any commercial or financial relationships that could be construed as a potential conflict of interest.

## Supplementary material

Supplementary Figure 1 Map of voxels with greater radial diffusivity (RD; family-wise error rate corrected 0.064 ≤ *p* ≤ 0.094) in mild traumatic brain injury (mTBI) participants compared to healthy control participants. The average white-matter skeleton is presented in green. Yellow voxels indicate voxels with a trend toward statistical significance. Surrounding voxels are filled with red for visual purposes only. Images are in neurological orientation and Z-coordinates are presented in MNI standard space.

Supplementary Figure 2 Map of voxels indicating a positive trend between axial diffusivity (AD) and Satisfaction with Life Scale (SWLS) total scores in the healthy control participants. The average white-matter skeleton is presented in green. Yellow voxels indicate positive correlations between AD and SWLS total score (family-wise error rate corrected 0.068 ≤ *p* ≤ 0.099). Surrounding voxels are filled in red for visual purposes only. Images are in neurological orientation and Z-coordinates are presented in MNI standard space.

Supplementary Figure 3 Map of voxels indicating a positive trend between mean diffusivity (MD) and Pittsburgh Sleep Quality Index (PSQI) total scores in the mild traumatic brain injury (mTBI) participants. The average white-matter skeleton is presented in green. Yellow voxels indicate positive correlations between MD and PSQI total score (family-wise error rate corrected 0.079 ≤ *p* ≤ 0.1). Surrounding voxels are filled in red for visual purposes only. Images are in neurological orientation and Z-coordinates are presented in MNI standard space.

Supplementary Figure 4 Map of voxels indicating a positive trend between axial diffusivity (AD) and Rivermead Post-concussion Symptom Questionnaire − 3 (RPQ3) scores in the mild traumatic brain injury (mTBI) participants. The average white-matter skeleton is presented in green. Yellow voxels indicate negative correlations between AD and RPQ3 total score (family-wise error rate corrected 0.093 ≤ *p* ≤ 0.1). Surrounding voxels are filled in red for visual purposes only. Images are in neurological orientation and Z-coordinates are presented in MNI standard space.

Supplementary Figure 5 Heatmap showing the distribution of labeled voxels (family-wise error rate corrected 0.05 < *p* ≤ 0.1). Anatomical labels are drawn from the JHU ICBM-DTI-81 White-Matter Labels atlas and retrieved using the FMRIB Software Library (FSL) *atlasquery* function. *Atlasquery* returns the probability (and, in the case of the JHU ICBM-DTI-81 atlas, the proportion) of voxels in a mask belonging to a region identified in a given atlas. The JHU ICBM-DTI-81 atlas does not encompass all white matter, and consequently some voxels remain unclassified. Colors reflect the percentage of labeled voxels identified within each anatomical location ($\frac{localized\;voxels}{\sum \left(classified\;voxels\right)}$. Black boxes indicate no voxels with a trend (family-wise error rate corrected 0.05 < *p* ≤ 0.1) were present in that anatomical location. BDI, Beck Depression Inventory; PSQI, Pittsburgh Sleep Quality Index; FA, Fractional Anisotropy; MD, Mean Diffusivity; RD, Radial Diffusivity. Anatomical location abbreviations are summarized in Supplementary Table 1.

Supplementary Table 1 JHU ICBM-DTI-81 White-Matter Labels atlas abbreviations.

Supplementary Table 2 Confirmatory *post-hoc* correlations.
