# Supplementary figures and images for: Diffusion Tensor Imaging (DTI) Correlates of Self-Reported Sleep Quality and Depression Following Mild Traumatic Brain Injury
Source: Front Neurol. 2018 Jun 20;9:468. doi: 10.3389/fneur.2018.00468 (PMC6019466; doi:10.3389/fneur.2018.00468)

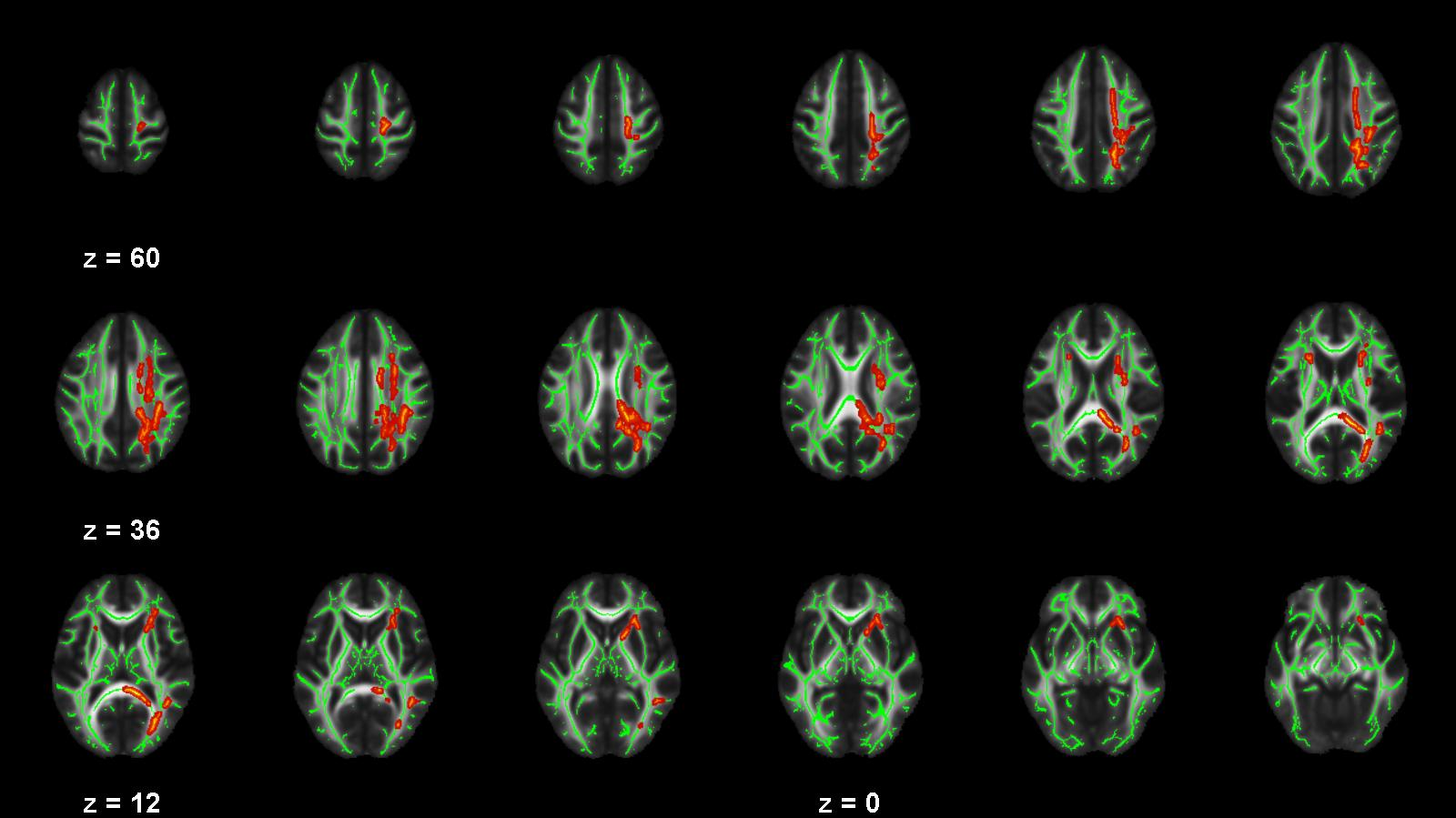

Supplement: Supplementary Figure 1 — Map of voxels with greater radial diffusivity (RD; family-wise error rate corrected 0.064 ≤ p ≤ 0.094) in mild traumatic brain injury (mTBI) participants compared to healthy control participants. The average white-matter skeleton is presented in green. Yellow voxels indicate voxels with a trend toward statistical significance. Surrounding voxels are filled with red for visual purposes only. Images are in neurological orientation and Z-coordinates are presented in MNI standard space. [file Image_1.TIF]

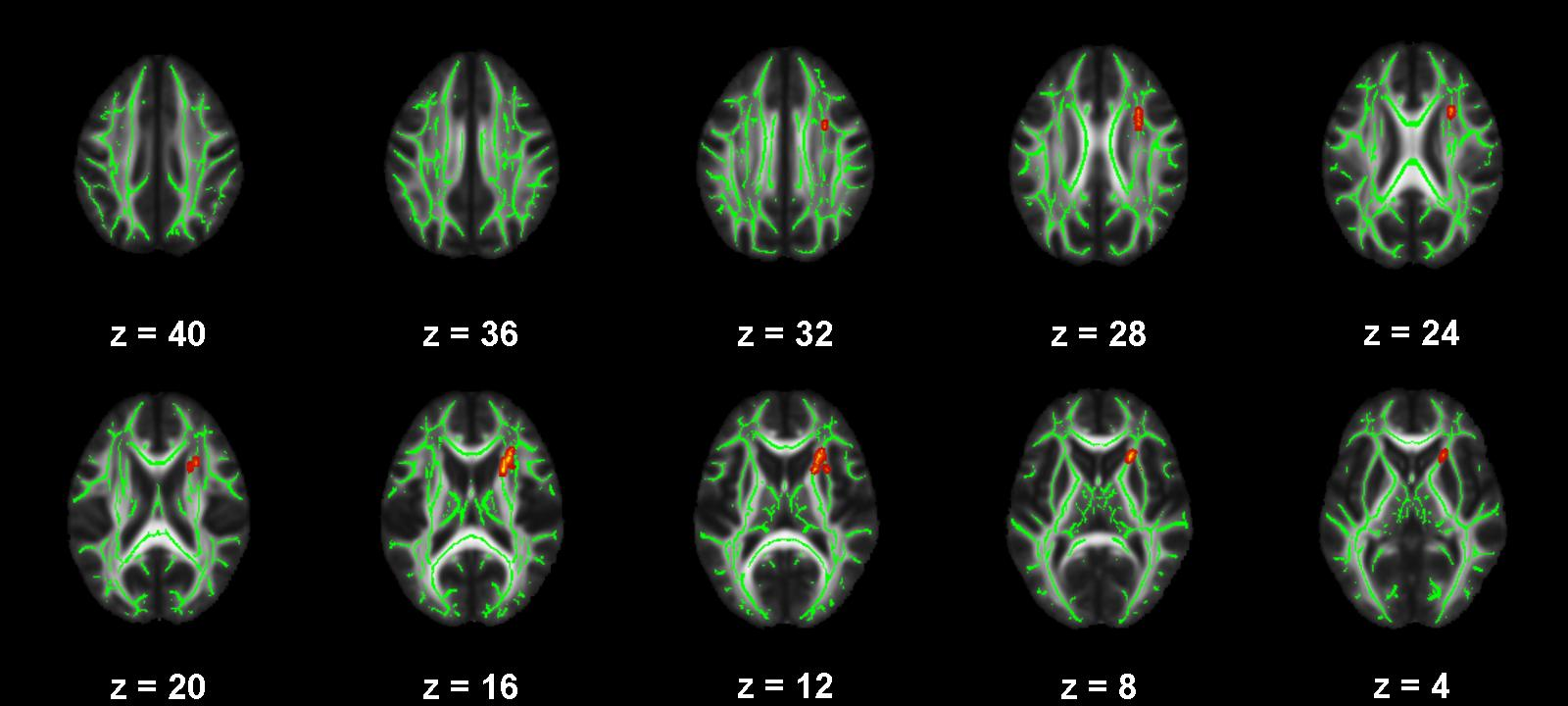

Supplement: Supplementary Figure 2 — Map of voxels indicating a positive trend between axial diffusivity (AD) and Satisfaction with Life Scale (SWLS) total scores in the healthy control participants. The average white-matter skeleton is presented in green. Yellow voxels indicate positive correlations between AD and SWLS total score (family-wise error rate corrected 0.068 ≤ p ≤ 0.099). Surrounding voxels are filled in red for visual purposes only. Images are in neurological orientation and Z-coordinates are presented in MNI standard space. [file Image_2.TIF]

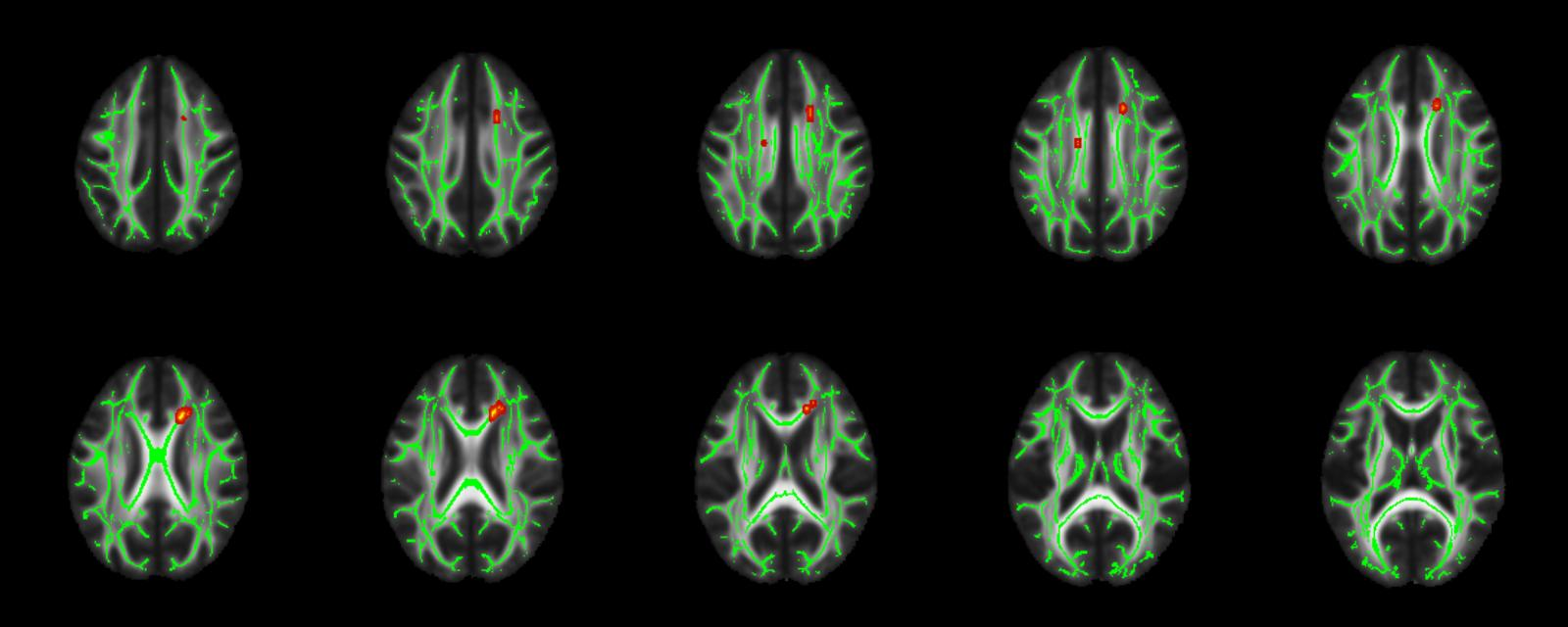

Supplement: Supplementary Figure 3 — Map of voxels indicating a positive trend between mean diffusivity (MD) and Pittsburgh Sleep Quality Index (PSQI) total scores in the mild traumatic brain injury (mTBI) participants. The average white-matter skeleton is presented in green. Yellow voxels indicate positive correlations between MD and PSQI total score (family-wise error rate corrected 0.079 ≤ p ≤ 0.1). Surrounding voxels are filled in red for visual purposes only. Images are in neurological orientation and Z-coordinates are presented in MNI standard space. [file Image_3.TIF]

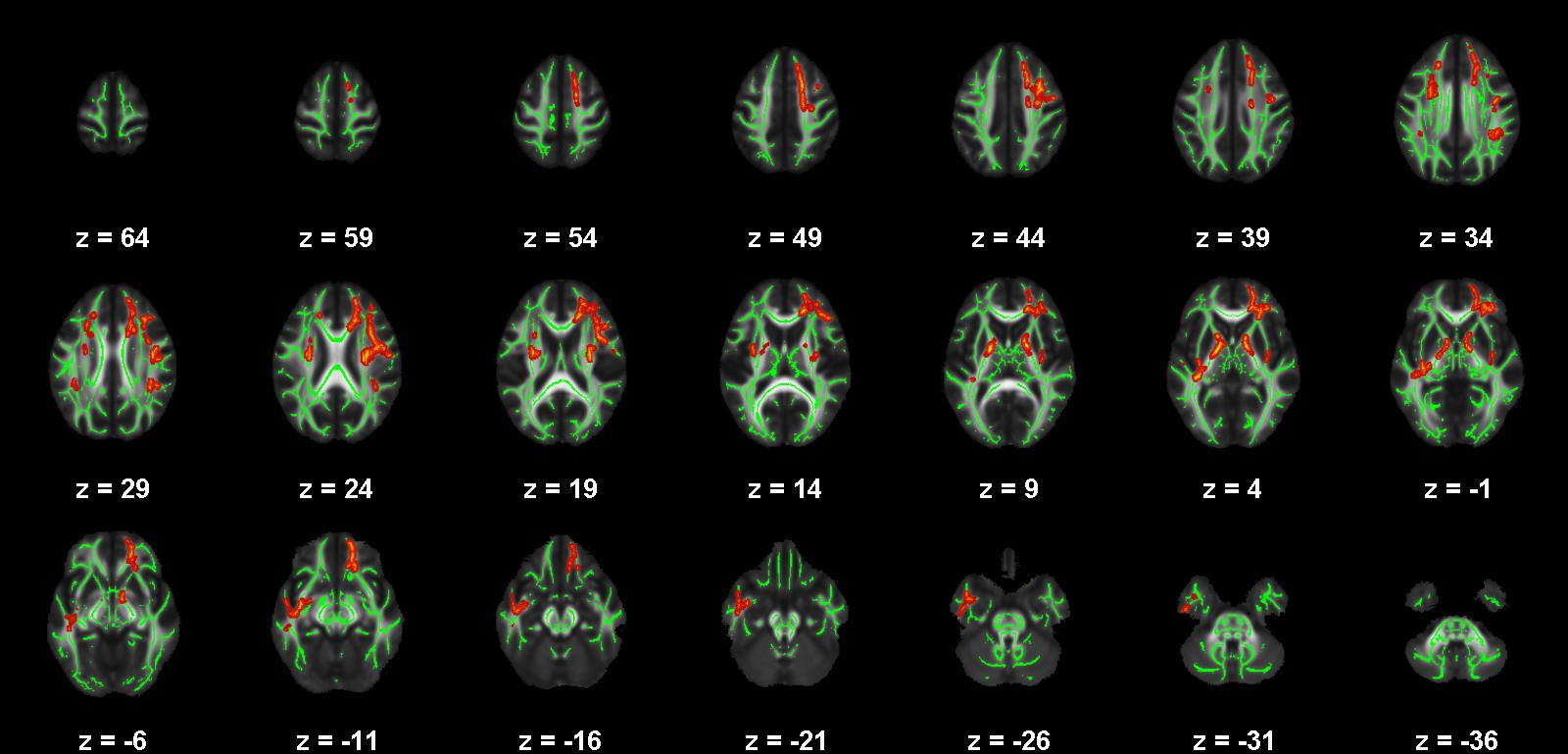

Supplement: Supplementary Figure 4 — Map of voxels indicating a positive trend between axial diffusivity (AD) and Rivermead Post-concussion Symptom Questionnaire − 3 (RPQ3) scores in the mild traumatic brain injury (mTBI) participants. The average white-matter skeleton is presented in green. Yellow voxels indicate negative correlations between AD and RPQ3 total score (family-wise error rate corrected 0.093 ≤ p ≤ 0.1). Surrounding voxels are filled in red for visual purposes only. Images are in neurological orientation and Z-coordinates are presented in MNI standard space. [file Image_4.TIF]

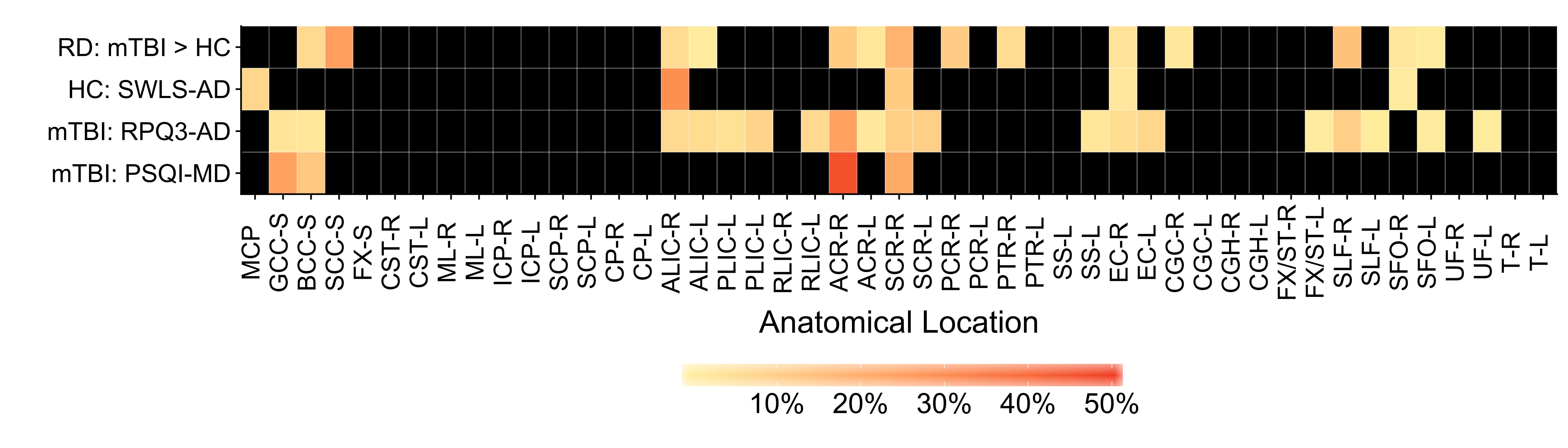

Supplement: Supplementary Figure 5 — Heatmap showing the distribution of labeled voxels (family-wise error rate corrected 0.05 < p ≤ 0.1). Anatomical labels are drawn from the JHU ICBM-DTI-81 White-Matter Labels atlas and retrieved using the FMRIB Software Library (FSL) atlasquery function. Atlasquery returns the probability (and, in the case of the JHU ICBM-DTI-81 atlas, the proportion) of voxels in a mask belonging to a region identified in a given atlas. The JHU ICBM-DTI-81 atlas does not encompass all white matter, and consequently some voxels remain unclassified. Colors reflect the percentage of labeled voxels identified within each anatomical location (localized voxels∑(classified voxels). Black boxes indicate no voxels with a trend (family-wise error rate corrected 0.05 < p ≤ 0.1) were present in that anatomical location. BDI, Beck Depression Inventory; PSQI, Pittsburgh Sleep Quality Index; FA, Fractional Anisotropy; MD, Mean Diffusivity; RD, Radial Diffusivity. Anatomical location abbreviations are summarized in Supplementary Table 1. [file Image_5.JPEG]
