# Supplementary material for: Diffusion Tensor Imaging (DTI) Correlates of Self-Reported Sleep Quality and Depression Following Mild Traumatic Brain Injury
Source: Front Neurol. 2018 Jun 20;9:468. doi: 10.3389/fneur.2018.00468 (PMC6019466; doi:10.3389/fneur.2018.00468)
Supplement: Supplementary Table 1 — JHU ICBM-DTI-81 White-Matter Labels atlas abbreviations. [file Table_1.DOCX]

| **Supplementary Table 1. JHU ICBM-DTI-81 White-Matter Labels atlas abbreviations** | | | | | |
| --- | --- | --- | --- | --- | --- |
| MCP | Middle cerebellar peduncle (Bilateral) | ALIC-L | Anterior limb of the internal capsule (Left) | EC-L | External capsule (Left) |
| GCC-S | Genu of corpus callosum (Bilateral) | PLIC-L | Posterior limb of the internal capsule (Right) | CGC-R | Cingulate gyrus (Right) |
| BCC-S | Body of corpus callosum (Bilateral) | PLIC-L | Posterior limb of the internal capsule (Left) | CGC-L | Cingulate gyrus (Left) |
| SCC-S | Splenium of corpus callosum (Bilateral) | RLIC-R | Retrolenticular part of the internal capsule (Right) | CGH-R | Cingulum (hippocampus) (Right) |
| FX-S | Fornix (column and body of fornix) (Bilateral) | RLIC-L | Retrolenticular part of the internal capsule (Left) | CGH-L | Cingulum (hippocampus) (Left) |
| CST-R | Corticospinal tract (Right) | ACR-R | Anterior corona radiata (Right) | FX/ST-R | Fornix (cres) / Stria terminalis (Right) |
| CST-L | Corticospinal tract (Left) | ACR-L | Anterior corona radiata (Left) | FX/ST-L | Fornix (cres) / Stria terminalis (Left) |
| ML-R | Medial lemniscus (Right) | SCR-R | Superior corona radiata (Right) | SLF-R | Superior longitudinal fasciculus (Right) |
| ML-L | Medial lemniscus (Left) | SCR-L | Superior corona radiata (Left) | SLF-L | Superior longitudinal fasciculus (Left) |
| ICP-R | Inferior cerebellar peduncle (Right) | PCR-R | Posterior corona radiata (Right) | SFO-R | Superior fronto-occipital fasciculus (Right) |
| ICP-L | Inferior cerebellar peduncle (Left) | PCR-L | Posterior corona radiata (Left) | SFO-L | Superior fronto-occipital fasciculus (Left) |
| SCP-R | Superior cerebellar peduncle (Right) | PTR-R | Posterior thalamic radiation (Right) | UF-R | Uncinate fasciculus (Right) |
| SCP-L | Superior cerebellar peduncle (Left) | PTR-L | Posterior thalamic radiation (Left) | UF-L | Uncinate fasciculus (Left) |
| CP-R | Cerebral peduncle (Right) | SS-L | Sagittal stratum (Right) | T-R | Tapetum (Right) |
| CP-L | Cerebral peduncle (Left) | SS-L | Sagittal stratum (Left) | T-L | Tapetum (Left) |
| ALIC-R | Anterior limb of the internal capsule (Right) | EC-R | External capsule (Right) |  |  |
| Note: Anatomical label abbreviations for the JHU-ICBM-DTI-81 atlas (Oishi et al., 2008) | | | | | |
