# Supplementary material for: Diffusion Tensor Imaging (DTI) Correlates of Self-Reported Sleep Quality and Depression Following Mild Traumatic Brain Injury
Source: Front Neurol. 2018 Jun 20;9:468. doi: 10.3389/fneur.2018.00468 (PMC6019466; doi:10.3389/fneur.2018.00468)
Supplement: Supplementary Table 2 — Confirmatory post-hoc correlations. [file Table_2.DOCX]

| **Supplementary Table 2. Confirmatory post-hoc correlations** | | | | |
| --- | --- | --- | --- | --- |
|  | | | | |
| ***Differences between correlation coefficients: Healthy Control vs. mTBI*** | | | | |
|  | Healthy Control^a^ | mTBI^b^ | Z | *p*^c^ |
| BDI-FA | -0.276^d^ | -0.758 | 2.252 | 0.024 |
| BDI-MD | 0.199^d^ | 0.791 | -2.774 | 0.006 |
| BDI-RD | 0.205^d^ | 0.798 | -2.814 | 0.005 |
| PSQI-FA | 0.285 | -0.805 | 4.470 | < 0.001 |
| PSQI-RD | -0.155 | 0.793 | -3.929 | < 0.001 |
|  |  |  |  |  |
| ***Differences between correlation coefficients:  Original BDI Total vs. Adjusted BDI Total (mTBI only)***^b,e^ | | | | |
|  | Original BDI | Adjusted BDI |  |  |
| BDI-FA | -0.758 | -0.739 | -1.208 | 0.227 |
| BDI-MD | 0.791 | 0.793 | -0.1365 | 0.891 |
| BDI-RD | 0.798 | 0.791 | 0.4814 | 0.630 |
| ^a^Partial correlation coefficient controlling for age and sex.  ^b^Partial correlation coefficient controlling for age, sex, and time post-injury  ^c^Tests are two-tailed z tests  ^d^BDI scores for the healthy control group were inverse transformed ($y= 1/{(x+1)}$) prior to computing the correlation to address skewness in the data. Positive correlations here indicate an inverse relationship, with higher BDI scores being associated with lower DTI values.  ^e^The correlation between the original and adjusted BDI was r = 0.991  mTBI: mild traumatic brain injury; BDI: Beck Depression Inventory | | | | |
